# Artificial Intelligence in Heart Failure: Friend or Foe?

**DOI:** 10.3390/life14010145

**Published:** 2024-01-19

**Authors:** Angeliki Bourazana, Andrew Xanthopoulos, Alexandros Briasoulis, Dimitrios Magouliotis, Kyriakos Spiliopoulos, Thanos Athanasiou, George Vassilopoulos, John Skoularigis, Filippos Triposkiadis

**Affiliations:** 1 Department of Cardiology, University Hospital of Larissa, 41110 Larissa, Greece; 2 Division of Cardiovascular Medicine, Section of Heart Failure and Transplantation, University of Iowa, Iowa City, IA 52242, USA; 3 Department of Cardiothoracic Surgery, University of Thessaly, 41110 Larissa, Greece; dmagouliotis@gmail.com (D.M.); spiliopoulos@uth.gr (K.S.); 4 Department of Surgery and Cancer, Imperial College London, St Mary’s Hospital, London W2 1NY, UK; 5 Department of Hematology, University Hospital of Larissa, University of Thessaly Medical School, 41110 Larissa, Greece

**Keywords:** artificial intelligence, heart failure, machine learning, limitations, ethical

## Abstract

In recent times, there have been notable changes in cardiovascular medicine, propelled by the swift advancements in artificial intelligence (AI). The present work provides an overview of the current applications and challenges of AI in the field of heart failure. It emphasizes the “garbage in, garbage out” issue, where AI systems can produce inaccurate results with skewed data. The discussion covers issues in heart failure diagnostic algorithms, particularly discrepancies between existing models. Concerns about the reliance on the left ventricular ejection fraction (LVEF) for classification and treatment are highlighted, showcasing differences in current scientific perceptions. This review also delves into challenges in implementing AI, including variable considerations and biases in training data. It underscores the limitations of current AI models in real-world scenarios and the difficulty in interpreting their predictions, contributing to limited physician trust in AI-based models. The overarching suggestion is that AI can be a valuable tool in clinicians’ hands for treating heart failure patients, as far as existing medical inaccuracies have been addressed before integrating AI into these frameworks.

## 1. Introduction

Heart failure (HF) is a long-term, advancing condition where the cardiac muscle debilitates due to various factors, leading to an insufficient supply of blood to meet the body’s oxygen demands [1]. As per research carried out by the European Society of Cardiology (ESC), HF impacts 26 million adults worldwide, with around 3.6 million new diagnoses being identified each year [2]. Artificial intelligence (AI) constitutes a computational program which possesses the ability to process functions which are deemed typical of human intellectual functions, such as the identification of certain patterns or images, programming, the recognition of sounds or objects and problem solving [3]. AI grants a device the ability to make autonomous decisions based on data which have been previously collected. In medical practice, this concerns data which are being used for diagnosis establishment or selecting the optimum treatment option.

In the contemporary era of evidence-based medicine, the medical community has access to a plethora of scientifically documented data upon which therapeutic options rely, while the imminent evolution ushers us to precision medicine. Concerning cardiovascular prevention and treatment, precision medicine accounts for a holistic approach which incorporates the summation of genetic factors, lifestyle and exposition to harmful factors in order to mold the phenotype of the cardiovascular status of each patient [4]. AI’s line of action goes beyond the apheretic perspective, in which it is arbitrarily deduced that patients with common disease traits share the same pathophenotype and will consequently be benefited by the same treatment [5]. AI’s role in precision medicine, and in the field of HF in particular, is to detect among this huge data set and recompose those elements which turn out to be advantageous and can correlate to a beneficial clinical outcome. 

In this review, we attempt to present an overview of the applications of AI in the field of HF, considering the limitations that thus far exist and restrict its clinical use.

## 2. Machine Learning

In order to be able to understand AI’s role in every day clinical practice, it is essential for healthcare professionals to familiarize themselves with some fundamental terms of AI. In the field of medicine, the vast majority of applications primarily concentrate on the learning aspect, employing machine learning (ML) as the primary methodology. ML encompasses a set of algorithms that acquire the ability to achieve a goal without the need for strict and definite programming [6,7]. During the procedure, the program learns and evolves from data that have been previously provided instead. This emerges in the scientific fields of statistics and computer science [8]. 

### 2.1. Supervised Learning

Supervised learning is mainly employed for tasks/problems of categorization and prognosis. A pre-signalized total set of data is used as a ground truth, which is used as a basis upon which the program learns to identify similar traits or algorithms in the new data set which is provided afterwards [9]. Supervised ML involves an examination of the data, in which labeled variables are carefully chosen, processed and assigned weights to determine the optimal combination for achieving the desired outcome. When dealing with intricate outcomes that demand expert assessments and annotations, researchers are required to provide labels for the original data [10]. This aspect can result in the supervised ML approach being occasionally more demanding in terms of time and resources compared to unsupervised ML. Supervised ML algorithms have demonstrated their effectiveness in precisely identifying HF when utilized with long-term electrocardiogram (ECG) time series data [11]. LASSO regression, Bayesian networks, AdaBoost, random forests, support vector machines (SVM) and artificial neural networks (ANN) represent some examples of supervised ML algorithms [12].

### 2.2. Unsupervised Learning

Unsupervised ML represents the ability of a program to evolve in order to accomplish a task based on algorithms that have previously been formed autonomously. It is designed to detect existing associations or patterns in a prespecified dataset without a label. In other words, the algorithm of clustering or association is figured out by the program based on traits detected in the dataset. This process facilitates the detection of disease mechanisms, phenotypes and genotypes in the supplied total of information [13]. An advantage of unsupervised learning and the absence of labeled data is that the algorithm pattern is set up leisurely, allowing for an inventive new grouping that humans had possibly not previously perceived. The new categorization formed by the computational program can, thereafter, be used for supervised learning in a dataset [14]. Unsupervised ML examples encompass partitioning algorithms such as K-means clustering and hierarchical clustering. Shah and colleagues, in their study, utilized 67 continuous input variables and an agglomerative hierarchical clustering algorithm to create a phenomapping of 397 patients with HFpEF. This phenomapping aimed to highlight distinctions in terms of cardiac structure, function, clinical characteristics, outcomes and hemodynamics among these patients [15]. 

### 2.3. Reinforcement Learning

Reinforcement learning is applied when a ML algorithm receives ongoing feedback from its environment in the form of reiterative information regarding the outcomes of its predictions. This feedback plays a crucial role in shaping and improving its future predictions [16,17]. Thus far, this approach has mainly been used within the domains of gaming and robotics. In this approach, the algorithm is formed through consecutive successes and failures in achieving a goal, which in turn raises ethical concerns in the case where there is no allowance for mistakes when human life is involved [18]. However, a previous investigation into protocolized sepsis care demonstrated the capability of reinforcement ML to integrate complex time-series data and provide insights into sequential treatment interventions [19]. 

### 2.4. Deep Learning and Convolutional Neural Networks

Deep learning (DL) represents a particular form of ML that leverages ANN for the automated generation of predictions directly from input data. In the realm of medical image analysis, convolutional neural networks (CNNs) are the most frequently employed DL networks [20]. The DCNN is consistently fine-tuned and improved, using the conventional neural network as a foundation [21]. It maintains a comparable hierarchical structure to the neural network but incorporates additional layers. Furthermore, each layer in the network conducts distinct processing, and there have been simplifications in the connections. Typically, a DCNN consists of fundamental components such as the data input layer, convolution layer, activation function, other processing layers and the output layer. The role of the input layer is to receive input information. Typically, this input layer is multidimensional and has the capacity to preserve the structural details inherent in the image data itself. The convolutional layer, on the other hand, employs convolution kernels to process input samples and extract features [22,23]. It is worth noting that the convolution kernel can change as the network’s depth increases. Through convolution, fundamental information is extracted, including features like lines and contours. The specific type of information drawn out is influenced by the location of the convolutional layer within the network; in other words, the deeper the convolutional layer, the broader its recipient plain becomes.

## 3. Applications of AI in HF

### 3.1. HF Diagnosis

The diagnosis of HF depends on a combination of patient history, clinical examinations, along with the evaluation of imaging and laboratory data. A delayed diagnosis can lead to a postponement in the administration of optimal medical treatment, which could potentially result in avoidable deaths or hospitalizations that might have been prevented if appropriate treatment had been initiated earlier. Present-day research involving AI-based models to improve the diagnosis of HF has encompassed various data sources such as electrocardiograms (ECG), echocardiography, X-rays and electronic health record (EHR) data [24,25,26,27,28,29,30]. These studies have demonstrated impressive performance assessments when using these sources. In a study of Masetic et al., ECG signals from two databases were utilized to construct a model employing the random forest method [31]. In both databases, the algorithms exhibited high precision, with HF detection rates falling within the range of 95% to 100%. When employing convolutional neural networks (CNN), both experiments showcased outstanding validity, also varying from 95% to 100% when using the random forest approach. However, the aforementioned datasets were confined to a subgroup of HF patients, as they did not include patients with a preserved ejection fraction (HFpEF).

A better validation of the deep learning technique in diagnosing HF is expected after the completion of the EAGLE (ECG-AI-Guided Screening for Low Ejection Fraction, NCT04000087) trial [32]. A deep learning algorithm using a 12-lead ECG has been developed and assimilated to the electronic health record in order to screen for HF with a reduced ejection fraction (HFrEF), while a following confirmatory echocardiogram will enable diagnosis and therapy. This will be one of the initial attempts to assess the practical utility of AI through a prospective evaluation in real-world scenarios.

A chest X-ray is typically the initial imaging method used because it is affordable, noninvasive and assists in distinguishing between the causes of dyspnea related to the heart and the lungs. Celik et al. analyzed the chest X-rays of 10.100 subjects of outpatient departments using an AI algorithm (Qxr) based on CNNs as a diagnostic tool [33]. Chest X-rays with a CTR > 0.5 and bilateral pleural effusion were flagged as potential HF X-rays. The corresponding patients underwent confirmatory tests to set or reject the diagnosis of HF. There were also subjects not marked as potential HF patients were who were randomly sampled and evaluated for HF diagnosis. Overall, the algorithm demonstrated a positive predictive value of 77% and a negative predictive value of 91%, performing well even in diagnosing HFpEF. Thus, 54% of the diagnosed patients had HFpEF.

### 3.2. HF Monitoring and Management with Sensors

Thanks to ongoing technological progress, sensors have become a vital inclusion in our daily lives, serving a role in nearly every common application we encounter. The types of sensors utilized in cardiovascular research are hemodynamic and biochemical sensors [34]. In the first category, CardioMEMS™, an implantable pulmonary artery pressure monitoring device, could assist in preventing HF decompensations, thereby significantly reducing HF hospitalizations [35]. A device designed to measure the pressure of the left ventricle was developed by the St. Jude Medical team. A wearable sensor was implanted on the intra-atrial septum of patients with HFrEF or HFpEF. Pressure guided therapy, according to the daily measurements derived, enabled physician-directed patient self-management and lead to a reduction in decompensation events and a significant fall in the mean left atrial pressure [36]. Biochemical sensors are devices that act as transducers, accepting bodily fluids as the input and supplying valuable data regarding the concentration of specific analytes and the status of the plasma volume [34]. During an invasive evaluation of HF patients, a dedicated sensor designed to measure both the venous oxygen saturation and right ventricular (RV) pressure was integrated. This method demonstrated considerable promise for potential use in future HF patients since it can assess two crucial parameters with a single sensor [37]. Another respiratory parameter that can provide valuable information about a HF patient’s status is minute ventilation. The identification of an individual with hyperventilation can help as an early indicator of HF decompensation and likewise enable timely treatment [38]. Combining wearable sensors with ML analytics has the potential to enhance results. In a recent study known as the LINK-HF (Multisensor Non-invasive Remote Monitoring for Prediction of Heart Failure Exacerbation) trial, ML analytics illustrated that remotely collected monitoring data, obtained non-invasively, can forecast HF rehospitalization with a sensitivity of 87.5% and specificity of 85% [39]. Mobile apps that incorporate ML algorithms have the potential to enhance HF care by motivating patients to embrace preventive tactics and adhere to their medication regimens.

### 3.3. ML-Based Prediction of Response to Cardiac Resynchronization Therapy

Howell et al. aimed to develop a prediction model for the short-term response to cardiac resynchronization therapy (CRT) in order to point out those HF patients who are suitable for early CRT implantation. A total of 741 patients with NYHA III-IV HF and EF < 35% pooled from the SMART-AV trial, were reviewed and multiple variables such as clinical, electrocardiographic, echocardiographic and biomarker characteristics were provided for eight different ML models [40]. The model achieved a prediction of the CRT response, with the primary end point being freedom from death, HF hospitalization and an LV end systolic volume index reduction > 15%, with an accuracy, sensitivity and specificity of 70%. This holds particular importance because having data available is crucial for making informed decisions for the important group of HF patients who stand to gain from a systematic approach to follow-up and interventions aimed at enhancing their end results.

In another study, Tokodi et al. utilized ML in order to evaluate sex-specific differences as predictors of mortality in CRT patients and to assess the prediction of one- and three-year mortality in the same patients. In total, 2191 CRT patients were evaluated using ML models in a retrospective study, with the results pointing out a significant discrepancy in the overall lifetime risk between males and females [41]. Specifically, the group of men featured a mortality rate of 35.2%, contradicting the 23.8% mortality rate in the group of women. The sex-specific variables predictive for mortality proved to be the NYHA functional class, LVEF and the etiology of HF for the group of women, while QRS morphology, hemoglobin levels and treatment with allopurinol had the most significance for the males.

In a retrospective trial of 1664 patients, Cai et al. attempted to identify CRT responders using contemporary methods of ML [42]. An ensemble of ensemble (EOE) method was the model of ML that was used in order to create supervised and unsupervised layers of stratification. They proposed that EoE performed better in discriminating patients with HF who were about to benefit from CRT implantation, compared to traditional ML methods and CNN models trained on ECG waveforms.

Gallard et al. conducted a retrospective trial, evaluating 323 patients with HF. The random forest (RF) technique was utilized to assess the importance of various features, identify the most crucial ones and construct an ensemble classifier [43]. The primary goal was to predict the response to CRT. The most considerable features to be highlighted was the QRS duration, septal flash, E/A, E/EA and left ventricular end diastolic-volume. The study results emphasize the importance of incorporating echocardiographic parameters in ML algorithms so as to enhance the effectiveness of prediction models concerning a CRT response [44]. 

## 4. Imaging HF with AI

### 4.1. Image Obtaining and Identification

Over the past ten years, advancements in image reconstruction processes and imaging technology have made significant strides in improving the quality and precision of medical images. This progress has led to reductions in the time needed for image acquirement and lowered radiation exposure [45]. These enhancements primarily involve the integration of data corrections into iterative reconstruction algorithms. However, one persistent challenge is the computational competence of these algorithms, particularly in the context of cardiovascular imaging [46,47].

While contemporary iterative algorithms produce high-quality images, they often struggle with rates of convergence, particularly when dealing with variations in the concentration of radionuclides in tissues. Researchers are actively working on further improving these algorithms to minimize radiation exposure and enable parametric imaging [48].

In contrast to other aspects of cardiovascular imaging, the utilization of deep learning (DL) for image reconstruction remains relatively uninvestigated. Most DL methods are either incorporated into traditional iterative reconstruction algorithms or directly applied to the raw scanner data. Some DL techniques enable direct image reconstruction from raw data and have been employed for tasks such as removing artifacts, correcting scattering and eliminating noise. This application is particularly notable in cardiac MRI, where it helps reduce motion artifacts [49,50]. 

Despite the potential of DL in tomographic reconstruction and data correction, there is a shortage of relative research and trials. This shortage of research hinders our competence to evaluate how reliable and precise DL methods are, correlated with the contemporary applied iterative reconstruction techniques across various clinical scenarios [51].

### 4.2. Image Segmentation

Image segmentation is one of the most well-established implementations of DL. An excellent illustration of a widely adopted segmentation network is the U-NET. It currently represents the cutting-edge segmentation algorithm utilized across various imaging modalities and clinical contexts [45]. It has demonstrated its substantial potential to emerge as the leading method in medical image segmentation, as evidenced by its strong performance in recent segmentation challenges [52,53]. 

### 4.3. AI-Based Echocardiography

There are already examples of AI being incorporated into echocardiographic evaluations either as a means of getting more accurate echocardiographic images and sparing valuable time of clinical practices, or with the purpose of targeting the correct diagnosis and the consequent results of the corresponding treatments, patients’ healthcare and hospitalizations [20].

As far as the quality of the obtained images are concerned, He et al. designed a trial including 3495echocardiographic reports in order to assess an LVEF evaluation based on AI [54]. They found that the initial assessment of LVEF by AI was non-inferior, but even superior to the assessment by sonographers, suggesting that AI can ameliorate efficacy and efficiency in assessing cardiac function.

In a prospective study mainly targeting the robustness of an AI-based diagnosis, Chen et al. evaluated 80 hospitalized patients with acute left HF. A deep convolutional neural network (DCNN) algorithm model was created in order to set up the image processing [55]. Patients were equally divided into the control group, undergoing a routine echocardiography, and the observation group, undergoing an echocardiography based on the DCNN model. After the comparison between the two groups, it was pointed out that the AI-based assessment demonstrated higher diagnostic accuracy and was related to lower rehospitalization and mortality rates. However, as the sample size was small, there was no statistical significance characterizing all the results.

Moreover, an AI-assisted echocardiographic evaluation could help address the unmet need for an accurate diagnosis in the large, heterogeneous group of patients with HFpEF. In a recent study of Stanford University, a deep learning model (Python, version 3.8.5) was employed in order to automate the echocardiographic assessment of patients, with attention mostly paid to measuring the left ventricular dimensions [24]. Pictures and videos displaying left ventricular hypertrophy were computationally assessed by a three-dimensional convolutional neural network with the purpose of distinguishing the causes of hypertrophy. The model was able to reliably identify cardiac amyloidosis and hypertrophic cardiopathy from other causes of LVH.

What is evident from the above is that AI-assisted echocardiographic interpretations can be applied retrospectively on an echocardiographic data basis in order to enhance our surveillance for undetected cases of relatively infrequent findings or early findings of dysfunction which may escape the attention of human interpreters. Measurements can be completely automated without lacking the reliability of the diagnosis at the same time. An automated approach for interpreting echocardiograms has the potential to promote accessibility to echocardiography, transferring heart assessments to primary care facilities and remote rural regions, thereby making it more widely available [55].

### 4.4. Cardiac MRI

Cardiac magnetic resonance (CMR) is acknowledged as the superior method for evaluating the cardiac structure and overall systolic function and is achieved by measuring precise blood and myocardial volumes [56]. The process of acquiring images is standardized and can be completed quickly. However, the analysis of these images is both time-consuming and reliant on the operator, leading to inherent variability between different observers and even within the same observer. Deep learning (DL) can offer a solution by automating this task with high levels of accuracy and precision. The protocols of AI for the automatic segmentation of the left ventricle with scars using LGE-MRI have already been developed, with promising results [57]. 

In a cohort study, strain CMR values generated from AI-based contours were compared with manually derived strain values. The protocol was set to assess CMR images from 136 subjects, including healthy volunteers and patients with LVH and myocardial infarction. According to the results, the AI-derived strain values performed well in healthy volunteer images and most patient groups with an overestimation of radial strain and underestimation of circumferential and longitudinal strain. The above research demonstrated that utilizing AI-generated contours for feature tracking and strain analysis in cardiac magnetic resonance (CMR) produces results that are on par with manual segmentations [58]. It is crucial to exercise caution when assessing cases of left ventricle hypertrophy, particularly in patients with aortic stenosis, regardless of whether manual or AI-generated contours are employed.

AI has also demonstrated potential in the field of CMR tissue characterization. Late gadolinium enhancement imaging is a crucial biomarker for assessing left ventricular scar volume, essential for risk assessment and procedure planning [59]. Deep learning convolutional neural networks (DL CNNs) can differentiate between left ventricular scar tissue and normal myocardium based on image-based characteristics. This capability allows for the precise measurement of scar volume in patients with ischemic and hypertrophic cardiomyopathies [59,60]. 

Finally, cardiac magnetic resonance (CMR) indicators can also be integrated into ML models to improve the accuracy of risk prediction. Ambale-Venkatesh and colleagues, as described in their study, utilized a random forest algorithm to demonstrate that among 725 clinical and imaging factors, the left ventricular structure and function derived from CMR imaging were identified as significant predictors of the onset of HF in a cohort of 6814 participants from the MultiEthnic Study of Atherosclerosis [61].

AI algorithms play a crucial role in cardiac imaging, particularly in ventricular segmentation using techniques like Cardiac MRI. ML can accurately segment heart chambers from Cardiac MRIs, providing imaging biomarkers for predicting congestive heart failure (CHF). Knowledge-based reconstruction has shown excellent accuracy in the 3D volumetry of the right ventricle. AI-assisted 3D visualization aids in diagnosing complex diseases, such as those affecting the right ventricle. Automated AI programs enhance accuracy in tasks like left ventricular mass calculation, papillary muscle identification and arterial measurements. Studies utilizing DL and SVM models with Cardiac MRI images consistently demonstrate their efficacy, as summarized in Table 1.

### 4.5. Nuclear Cardiology

In recent years, there have been notable developments in the use of AI applications within the field of nuclear cardiology. AI can already be implemented in SPECT/CT and PET/CT, mainly for the automation of image detection and selection and the risk assessment and diagnosis of CAD [45]. MPI data have also been integrated into ML models to predict outcomes. In an examination of the REFINE-SPECT registry, which comprises over 20,000 patients undergoing stress myocardial perfusion imaging (MPI), conducted in multiple international centers, Hu and colleagues applied ML techniques. They combined quantitative assessments of regional perfusion deficits with stress test results and clinical data to forecast the likelihood of early coronary revascularization [74,75]. Their results demonstrated that ML predictions surpassed both clinician interpretations and stress TPD (total perfusion deficit) assessments. However, there is yet scarce evidence of AI implementation in nuclear cardiology tests for patients with HF.

## 5. Main Limitations in AI’s Application

What has been called ‘the Achilles’ heel of AI is its consequent production of incorrect or inaccurate results after supplying the ML system with erroneous data, or the ‘garbage in, garbage out’ (GIGO) process. It is widely recognized that when AI is trained using relativist and prejudiced data, there is a potential for systematic errors to occur. Nonetheless, even AI applications that have been trained impeccably can generate incorrect results when provided with inaccurate input [74,76]. Regrettably, it would be rather unrealistic to assume that we could avoid providing the system with faulty importation regarding HF diagnosis and treatment, since there is abundant evidence advocating that our (level of) established knowledge is still inadequate. Issues that remain unresolved concern diagnosis, classification and treatment; consequently, AI outcomes should always be interpreted with caution.

### 5.1. HF Diagnosis

**Figure 1:**
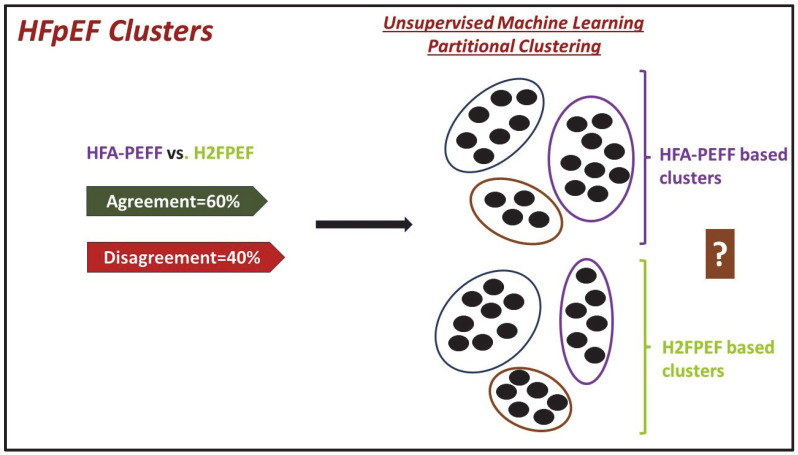
Example of perpetuation or even augmentation of diagnostic uncertainties with machine learning. The two currently used algorithms for HFpEF diagnosis are HFA-PEFF and HF2FPEF. These two algorithms disagree in approximately 40% of the cases. Thus, the HFpEF clusters obtained with the use of unsupervised machine learning will strongly depend on which algorithm was used and the diagnostic uncertainty will culminate in cluster definition uncertainty. A prerequisite, therefore, to proceed with unsupervised machine learning is an accurate clinical diagnosis, otherwise GIGO (Garbage In, Garbage Out); HFpEF, heart failure with preserved ejection fraction; HFA-PEFF, Heart Failure Association Pre-test assessment, Echocardiography and natriuretic peptide, Functional testing, Final etiology; H2FPEF, Heavy, 2 or more Hypertensive drugs, atrial Fibrillation, Pulmonary hypertension, Elder age > 60, elevated Filling pressures. Based on data from Ref. [78].

Until now, there have been two published algorithms for HFpEF diagnosis: H2FPEF and the European Society of Cardiology HFA-PEFF [77]. The ESC HFA-PEFF score shares certain variables with the H2FPEF score, but incorporates additional parameters leading to the anticipation of variations in the prevalence of HFpEF depending on the scoring system employed. Given the above discrepancies, disagreements may be present regarding which score should be utilized by the ML system in order to diagnose HFpEF (Figure 1).

### 5.2. LVEF-Based HF Classification

The classification of HF according to LVEF has been raising concerns. In the scientific community, disagreements exist regarding the physiological significance of LVEF, as well as LVEF cutoffs. According to the American Society of Echocardiography and the European Association of Cardiovascular Imaging, the normal LVEF range is 52–72% and 54–74% in men and women, respectively [79]. Nonetheless, recent findings challenge this classification as researchers have proposed a U-shaped relationship for LVEF and all-cause mortality, since hazard rates seem to increase in LVEF ranges higher than 65% according to several studies [80,81]. Equally troubling is the unclear physiologic significance of LVEF. LVEF, which is calculated as the LV stroke volume divided by the LV end-diastolic volume (LVEDV) and is typically expressed as a percentage, is erroneously presumed to be an indicator of myocardial contractility. It is important to note, however, that LVEF is influenced by loading conditions and cannot accurately represent myocardial contractility without accounting for LV loads. Furthermore, structural changes that lead to alterations in LVEDV can significantly affect LVEF, regardless of the actual level of contractility and stroke volume [82,83]. Based on the above, many experts question LVEF as a valid indicator for HF classification.

### 5.3. HF Treatment

HF treatment is currently based on LVEF, although there is an existing inconsistency between ESC and ACC/AHA guidelines and there are arguments advocating abandoning LVEF for treatment guidance. American HF guidelines have stressed the importance of addressing hypertension as a primary and significant aspect of drug therapy for HF, whether patients have a reduced ejection fraction (HFrEF) or preserved ejection fraction (HFpEF) [84]. As a result, the use of ACEIs or ARBs and the widespread adoption of MRAs for HF, regardless of the left ventricular ejection fraction (LVEF), have generally constituted appropriate pharmaceutical treatments in the United States. However, European HF guidelines have shown less emphasis on addressing concurrent conditions like hypertension, leading to some debate [85]. There is uncertainty regarding whether patients with HFpEF are currently receiving fundamental drug therapies or will do so in the future. Evidence indicates that clinical trials evaluating medication in HFpEF patients failed to present a benefit for various and distinct reasons, other than the medication tested being actually ineffective. Medication tested for HFrEF patients such as ACEIs, ARBs and MRAs was already established as background therapy for hypertension, making the effort to document the same beneficial effects in HFpEF seem ironic [86]. Furthermore, the acknowledgment of HFpEF as a distinct medical condition occurred relatively late in the duration of patent protection for certain medications which had established advantages and were consequently administered to patients with HFrEF [87,88]. As a result, except for a few cases, the pharmaceutical sector did not sponsor randomized controlled trials involving drugs that had lost or were about to lose their patent protection.

Taking in mind the above aspects, one could assume that effort should firstly be made to correct the contemporary incorrect approaches before grounding AI algorithms in them, which could just amplify current mistakes. Despite advancements, the application of AI has remained constrained by ongoing challenges. Problems arise both in the cases of an excessive number of variables and in the scarcity of them. In the first case, an overabundance of variables can lead to a decline in model performance. Conversely, if there is a shortage of predictive features, ML algorithms might result in overfitting, which restricts their generalizability [89,90]. To mitigate these limitations, one approach is to perform validation on various independent datasets or discrete data sources. Without such precautions, prediction models might mistakenly correspond to idiosyncrasies in the dataset used for development.

An AI model’s efficacy depends on the quality of its training data. Machine learning has three types: supervised, unsupervised and reinforcement [91]. Supervised learning predicts known outcomes from labeled datasets, suitable for classification and regression, but demanding substantial data and time. Unsupervised learning uncovers hidden patterns without human guidance, but may face challenges with biased training datasets and difficulty in identifying initial cluster patterns, leading to potential inaccuracies. Implementing AI in heart failure faces a significant challenge: determining the best approach for integrating available data into the clinical workflow.

Another primary initial hurdle when implementing ML models revolves around the disparity in expectations and the inadequate alignment between the current capabilities of ML systems and their intended applications. In the buildup to the current excitement surrounding ML in the field of medicine, numerous assertions about the capabilities of these systems have been made, some of which were either overly optimistic or, at the very least, ahead of their time. Nevertheless, it is crucial to acknowledge that in its present state, ML largely represents an extension of conventional modeling methods, each with its unique constraints [6]. A further constraint arises when ML models exhibit a subpar performance beyond their training settings. Clinical prediction models are typically deployed in controlled environments with carefully systematized data and minimal application risks. However, when these ML models are explicated in real-world scenarios, they are often carried out significantly worse than their originally published performance suggests. The following factor relates to the challenge of understanding the predictions made by numerous ML techniques. In the case of many ML methods, including those relying on neural networks, no straightforward means exist to trace how the model arrives at a prediction based on the provided input features. Limited interpretability is frequently put forward as the primary reason why some physicians convey limited reliance on AI-based prediction models.

## 6. Ethical Considerations

Ethical concerns frequently impose restrictions on ML-based models. Systemic biases have impeded the inclusion of under-represented minority groups in ML algorithms. Developing ML algorithms using an incorrect collection of values not only hampers the efficacy of ML as a diagnostic and treatment implement due to its limited applicability, but also carries the risk of sustaining systematic inequalities in the general public [92]. As supervised ML models necessitate labeled datasets to function, a substantial effort must be expended by humans in the data labeling process. This increased human involvement raises the likelihood of inserting bias into the prognosis. Likewise, biases in the election, verification and sampling processes could also influence the prognostication made by AI when employed in clinical contexts [93].

## 7. Conclusions

ML and AI have the capacity to bring advantages to both patients and cardiologists. However, their potential can only be fully realized if clinicians actively participate in the integration and utilization of these innovative algorithms. This is essential due to the exponential growth in patient data, which has become increasingly complex, often involving numerous interconnected parameters. Furthermore, we should always bear in mind that convolutional algorithms cannot surpass human ability to predict, diagnose or treat HF since the system’s ability to do so is resulting from the quality of the dataset and the relevance of the information we have supplied it with. Similar to a fundamental characteristic of human wisdom, which entails finding an equilibrium between skepticism and knowledge, the quest for artificial wisdom through computational methods should stay non-dogmatic, cognizant of its boundaries, inquisitive and, thus, engaged in the exploration of fresh knowledge.

## Figures and Tables

**Table 1:** Application of machine learning, deep learning and support vector machine models using cardiac MRI-derived images.

N	Authors	Results
1	Avendi et al. [62]	Cardiac MRI datasets have the potential to be employed in creating a deep learning algorithm for the segmentation of the right ventricle.
2	Dawes et al. [63]	Supervised learning of 3D cardiac motion patterns predicts death and adverse outcomes in pulmonary disease patients.
3	Puyol-Antón et al. [64]	Combining MRI and echo datasets achieves a diagnostic accuracy of 94% for dilated cardiomyopathy.
4	Bernard et al. [65]	Employed Convolutional Neural Networks (CNNs) for the automated segmentation of the left ventricle.
5	Luo et al. [66]	Utilized Multi-view Convolutional Neural Networks (CNNs) to predict left ventricular ejection fraction using cardiac MRI images.
6	Bratt et al. [67]	Demonstrated the superiority of CNN-based segmentation over conventional methods through empirical evidence.
7	Kong et al. [68]	Employed cardiac MRI to identify the end-systolic time point.
8	Schlemper et al. [69]	Demonstrated the superiority of a series of CNNs over undersampled dynamic cardiac MRI.
9	Laser et al. [70]	Noted that knowledge-based reconstruction exhibits excellent accuracy in right ventricular 3D volumetry.
10	Karim et al. [71]	Algorithms exhibit superior overlap with the consensus ground truth compared to the majority of n-SD fixed-thresholding methods, except for the Full-Width-at-Half-Maximum (FWHM) fixed-thresholding method.
11	Kurzendorfer et al. [72]	Fractal Analysis and Random Forest Classification quantification method surpasses the x-fold standard deviation approach in performance.
12.	Fahmy et al. [73]	Deep learning’s efficacy in automatically segmenting the left ventricle and scar volume in hypertrophic cardiomyopathy, with strong agreement compared to manual segmentations.

## Data Availability

Not applicable.
